# Left ventricular function in acute inflammatory peri-myocardial diseases – new insights and long-term follow-up

**DOI:** 10.1186/1476-7120-10-42

**Published:** 2012-11-05

**Authors:** Marina Leitman, Vladimir Tyomkin, Eli Peleg, Laurian Copel, Zvi Vered

**Affiliations:** 1 Department of Cardiology, Assaf Harofeh Medical Center, Zerifin, 70300, Israel; 2 Department of Radiology, Assaf Harofeh Medical Center, Zerifin, Israel; 3 Sackler School of Medicine, Tel Aviv University, Tel Aviv, Israel

**Keywords:** Pericarditis, Myocarditis, Left ventricular function

## Abstract

**Background:**

Until recently acute inflammatory peri-myocardial syndromes have been associated with global rather regional left ventricular (LV) dysfunction. Recent advances in cardiac imaging with echocardiographic techniques and magnetic resonance imaging (MRI) permit comprehensive evaluation of global and regional LV function. Our study was aimed to assess regional LV function in 100 patients with acute perimyocarditis, and correlate these findings with the clinical presentation.

**Methods:**

We report on 100 patients with acute perimyocarditis admitted during 2008–2011, in whom LV function was assessed by semi-quantitative wall motion score analysis on conventional echo. Long-term mortality and recurrent hospitalization were also assessed.

**Results:**

Wall motion score in 100 patients with acute perimyocarditis demonstrated a significant predominance of regional wall motion abnormalities in the infero-postero-lateral LV wall. These data correspond well with speckle tracking results of a subgroup of these patients published earlier. Recent MRI data show frequent late enhancement of contrast in the infero-lateral region of the LV in patients with perimyocarditis. These observations were useful in re-classification of our patients into one of the following groups: pure or predominant pericarditis, and pure or predominant myocarditis. Over a mean period of 37 months, there was no mortality. Though recurrent hospitalizations were rather frequent, no significant differences were observed among groups.

**Conclusions:**

Regional wall motion abnormalities in the infero-postero-lateral segments of the LV are frequent in patients with acute perimyocarditis. Detailed echocardiographic examination early in the course of the disease should become a major factor in the clinical differentiation among the various clinical presentations of acute inflammatory peri-myocardial syndromes. The long-term outcome of these patients appears to be benign, though recurrent hospitalizations are not infrequent.

## Background

The clinical spectrum of acute inflammatory peri-myocardial syndromes is diverse. Diagnostic features include typical clinical presentation with chest pain aggravating with inspirium and supine position and ameliorating while sitting, often in the setting of a recent viral infection. Additional features include typical electrocardiographic changes, positive inflammatory markers and elevated cardiac enzymes (troponin (Tn). Conventional echo examination may identify pericardial involvement/effusion and myocardial dysfunction.

The guidelines on the diagnosis and management of the peri-myocardial diseases use the term pericarditis, with remark, that pericarditis is often accompanied by some degree of myocarditis, evidenced by global or regional myocardial dysfunction, elevations of troponins I and T, MB creatine-kinase or serum myoglobin levels [1]. The concept, that pericarditis and myocarditis share the same etiologic agents, and are the two sides of the same disease, was developed by Imazio [2,3] and is accepted now in clinical practice. In most cases of pericarditis mixed pericardial and myocardial involvement is present [2]. The presence of wall motion abnormalities and elevated troponin have been the hallmark of concomitant myocardial involvement. The range of acute inflammatory peri-myocardial syndromes is from "pure" pericarditis toward increased myocardial involvement through predominant pericardial involvement, predominant myocardial involvement, and "pure" myocarditis [2,3]. In light of this concept, we assessed a series of 100 patients with acute inflammatory peri-myocardial syndromes. We analyzed global and regional myocardial function in these patients and correlated these results with the clinical presentation, laboratory analysis and clinical course.

## Methods

We analyzed the medical history and echocardiographic data of 100 patients admitted to our hospital with the diagnosis of acute peri-myocardial inflammatory syndromes: pericarditis, perimyocarditis and myocarditis, during 2008–2011 according to hospital codes. Detailed history, clinical, laboratory, echo data were analyzed thoroughly and appropriateness of diagnosis was revised in every case. Diagnostic criteria included: Typical chest pain, recent upper respiratory tract infection, electrocardiographic changes, elevated cardiac enzymes, elevated CRP or ESR; EF, wall motion abnormalities and presence of pericardial effusion; results of coronary angiography and cardiac CT in patients with equivocal clinical picture and risk factors for coronary artery disease. According to these criteria we sought to include each patient into one of the 4 groups: *pericarditis*, *myocarditis*, *predominant pericarditis*, *predominant myocarditis*. Patients with *pericarditis* were selected according to the typical chest pain, normal or near normal ejection fraction, normal or near normal troponin level. Patients with minimal regional wall motion abnormalities, normal ejection fraction (>55%) and troponin level were also included in this group.

Patients with *predominant pericarditis* needed to have preserved global LV function (LVEF 50-60%) and elevated cardiac enzymes [Troponin/CPK].

Mild global LV dysfunction (LVEF 40-50%) and regional wall motion abnormalities were characteristic of patients with *predominant myocarditis*.

Patients with *myocarditis* presented with significant LV dysfunction (LVEF < 40%), extensive wall motion abnormalities and elevated cardiac markers were hallmarks of this group.

Wall motion score index was calculated based on the formula recommended by the American Society of Echocardiography, Schiller NB et al. [4]: WMSi = Sum of wall motion scores/number of segments visualized. 18 cardiac segments were used instead of the original 16 based on our previous studies [5,6].

Mean segmental wall motion score index, based on all echocardiographic examinations, was calculated for every cardiac segment. These results were presented as a color coded map [5,6].

Based on hospital records and data obtained from the Ministry on Interior Affairs, long-term mortality and recurrent hospitalizations were assessed.

## Results

### Clinical, demographic, epidemiologic and laboratory data of the patients

Mean age was 34.8 ±12 years, 85 were males, 95 of the 100 patients presented with typical chest pain. Typical diffuse ST segment elevation on ECG was observed in 88 patients. The rest had less typical ECG changes. Cardiac enzymes [Troponin/CK] were elevated in 62 patients, inflammatory markers CRP\ ESR were elevated in 98 patients. Pericardial effusion was present in 29 patients, in 8 of them it was moderate to large.

Coronary artery disease could not be excluded clinically in 21 patients and they underwent either coronary angiography or cardiac CT. None had significant coronary disease.

A detailed echocardiogram was performed during the admission in all patients. Mean EF = 55.4±7.6%, wall motion abnormalities were detected in 69 of the 100 patients.

Among the 100 patients (Table 1), 2 patients were classified as *myocarditis*, 37 patients as *pericarditis*, 49 patients - *predominant pericarditis* and 12 patients - *predominant myocarditis*.

**Table 1:** Clinical characteristics of 100 patients with acute peri-myocardial inflammatory syndromes

	**Pericarditis**	**Predominantly pericarditis**	**Predominantly myocarditis**	**Myocarditis**
Number	37	49	12	2
Age	41+/-14	31+/-9	33+/-12	40+/10
Male /Female	29 /8	46/3	9/3	1/1
CRP / ESR	37 (100%)	All but 1(NA)	11** (91.7%)	2 (100%)
Tn / CK	N/Near N	49 (100%)	11 (91.7%)	2 (100%)
Catheterization/ Cardiac CT	1 (2.7%)	12 (24.5%)	7 (58.3%)	1 (50%)
Chest Pain	37(100%)	47 (95.9%)	10 (83.3%)	1 (50%)
ST elevation	32 (86.5%)*	44 (89.8%)	11 (91.7%)	1 (50%)
EF,%	59.3+/-1.6	56.4+/-5	44.2+/-7.2	27.5+/-10.6
Any WMA	14 (37.8%)	41 (83.7%)	12 (100%)	2 (100%)
WMA Apex	0	3 (6.1%)	1 (8.3%)	2 (100%)
WMA I-P-L	14 (37.8)	33 (67.3%)	5 (41.7%)	2 (100%)
WMA mixed	0	5 (10.2%)	6 (50%)	2 (100%)
Per. Effusion	17 (45.9%)	11 (22.4%)	1 (8.3%)	0
Respiratory infection, sore throat	21 (45.9%)	40 (71.4%)	6 (50%)	100%

* - 5 with less typical ECG changes had symptoms of recent onset, **-CRP on discharge.

*WMA* Wall motion abnormalities, *I-P-L* infero-postero-lateral, *URI* upper respiratory infection, *CK* creatin phosphokinase, Cardiac, *CT*–Coronary computed tomography, CRP C-reactive protein, *Tn* Troponin I, *ESR* erythrocyte sedimentation rate, *N* normal.

The 2 patients with *myocarditis* had severe LV dysfunction, extensive wall motion abnormalities, elevated cardiac enzymes and inflammatory markers.

Patients with *pericarditis* had normal global LV function (mean LVEF = 59.3 ±1.6%), normal or near normal regional LV function, normal or near normal troponin level, 32 of 37 of these patients had typical ST segment elevation on ECG. 5 patients with less typical ECG changes, arrived several days after the onset of symptoms. Pericardial effusion was present in 17 of them.

All 49 patients with *predominant pericarditis* had elevated cardiac enzymes, 44 had ST elevation on ECG, mean LVEF was lower than in patients with *pericarditis* 56.4 ±5 vs 59.3 ±1.6%, p<0003, 41 had wall motion abnormalities. Pericardial effusion was found in 11.

Among 12 patients with *predominant myocarditis*, cardiac enzymes and inflammatory markers were elevated in 11. Mean EF was 44.2±7.2%, and wall motion abnormalities occurred in all patients. Patients with *pericarditis* were significantly older than those with *predominant pericarditis:* 40.6±13 versus 30.6±9 years, p<0.0001. A trend toward older age was observed in the patients with *pericarditis* versus those with *predominant myocarditis* 41±14 versus 33±12, p=0.089.

### Wall motion abnormalities

**Figure 1:**
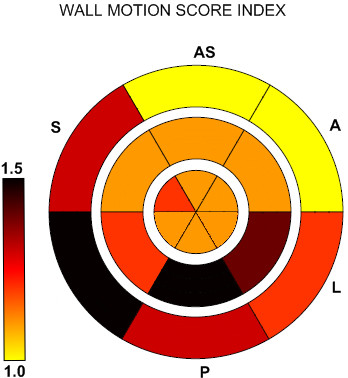
**Color coded map of the wall motion abnormalities over the left ventricle in 100 patients with perimyocarditis.** A – anterior, AS – antero-septal, S – septal, I –inferior, P-posterior, L – lateral wall of the LV. Outer ring corresponds to the basal segments, inner ring corresponds to the apical segments, and median ring is mid ventricular level of the LV. Wall motion abnormalities localized predominantly in the infero-basal and mid postero-lateral segments.

Involvement of the infero-postero-lateral segments occurred in 52 of 69 patients with regional wall motion abnormalities. In 6 patients apical and septal segments were involved, and in 13, concomitant involvement of infero/postero-lateral and septo-apical segments occurred. Color coded map [5,6] of mean segmental wall motion score index based on all echocardiographic exams of all the patients is presented in Figure 1: Regional wall motion abnormalities are localized predominantly in the infero-basal and mid postero-lateral segments.

A typical echo exam of a patient wih peri-myocarditis shows mid- posterior and mid-inferior hypokinesis [Additional file 1: Video].

### Outcome

Mean follow-up was 37±10 months [17–55 months]. During this period no mortality occurred in this group. Recurrent admission due to pericarditis was reported in 2 patients in *pericarditis* group [5.4%], 7 cases of recurrency in 6 patients with *predominant pericarditis* [14.3%], 5 cases in 3 patients in *predominant myocarditis* group [41.7%]. In the *myocarditis* group no recurrency was reported. There was no statistically significant difference in the recurrency rate between groups.

Recurrency rate over the entire 100 patients was 14 events [14%] in 11 patients.

One patient, 31 yo male with predominant myocarditis, had an ST elevation infero-posterior myocardial infarction 3 years after the original hospitalization treated by primary PCI.

## Discussion

The current report represents detailed analysis of patients with acute inflammatory peri-myocardial diseases evaluated by echocardiography. The majority of our patients were young, predominantly males. Older age of the patients judged to have *pericarditis* versus those with *predominant pericarditis* may be related to exaggerated immune response in younger patients.

Our data show, that most of the patients presented with the clinical picture of acute inflammatory peri-myocardial syndrome have mixed pericardial and myocardial involvement with progressive decrease of ejection fraction from normal in those with *pericarditis* to severe LV dysfunction in pure *myocarditis*.

Most of the patients had ST segment elevation on the ECG. The 5 patients with *pericarditis* and less typical ECG changes, had symptoms of recent rather than acute onset. Prevalence of pericardial effusion decreased from pure *pericarditis* to *predominant myocarditis*. Inflammatory markers, CRP or ESR, were elevated in most patients. Our results highlight the predominance of infero-postero-lateral segmental involvement in patients with pericardial inflammatory syndromes, and underscore the important role of careful analysis by conventional echo. Even in patients with apparently pure pericarditis and normal or near normal EF, we found involvement of the infero-postero-lateral segments in 38%.

Several mechanisms have been proposed: In a recent study by Mahrholdt et al. [7], the type of myocardial involvement, initial presentation and clinical course of 87 patients with biopsy proven myocarditis could be linked to the infective agent. Parvovirus PVB19-infected patients sought medical attention early because of severe chest pain, the EF was only mildly impaired (mean 55%), and late gadolinium enhancement on MRI was localized predominantly in the lateral wall of the LV. In herpes virus HHV6 myocarditis, clinical symptoms at initial presentation were more variable as well as LVEF (mean 42%). Symptoms of heart failure were present in half of HHV6 infected patients, late enhancement of gadolinium was most frequently found in the the anteroseptal region. The majority of patients with combined PVB19/HHV6 myocarditis presented with subacute onset of heart failure, severe LV dysfunction (mean LVEF 25%), and late enhancement in the entire septum. Subepicardial location of late contrast enhancement foci in acute myocarditis can be related to the close proximity of this region to the inflamed pericardium. Cardiotropic viruses including PVB19 may cause polyserositis and pericarditis after the initial viremia [7]. In spite of these fascinating data, the yield of viral serology is still uncertain, and it is not commonly recommended for the diagnosis of perimyocarditis [8]. In our series etiologic agent was not identified.

Myocarditis often mimics myocardial infarction, and in these patients coronary imaging is necessary to rule out significant coronary artery disease. In our series 21 of the 100 patients underwent coronary angiography or cardiac CT, all had normal or non-significant coronary artery disease. Coronary spasm in myocarditis has been previously proposed [9-11], but cases of true coronary spasm proven on coronary angiography in patients with acute myocarditis/pericarditis are rare [12,13]. In a recent study [14] biopsy proven PVB19 myocarditis was strongly associated with coronary spasm induced by acetylcholine testing during coronary angiography. The possible mechanism, proposed by the authors, is endothelial dysfunction induced by the myocardial inflammation or persistence of virus.

### Speckle tracking imaging in acute inflammatory pericardial disorders

Speckle tracking imaging – advanced echocardiographic technique, was developed recently for quantitative evaluation of left ventricular function in patients with ischemic heart disease, valvular heart disease and other myocardial disorders.

Recently, we have reported on a group of 38 patients with acute inflammatory pericardial syndromes, a sub-group of the current 100 patients, who were also assessed with speckle tracking imaging [6]. The results of that study strongly support the current data.

### Cardiac MRI in perimyocarditis

Although these observations highlight the importance of echocardiographic techniques in perimyocarditis, no doubt MRI should be considered the "gold standard" (15–18) : Mahrholdt et al. [15] in 2004, found delayed enhancement on MRI in patients with acute myocarditis (mean LVEF=47%) presented as a patchy distribution predominantly in the subepicardial region on the lateral free wall of the LV. In 21 of 28 patients biopsy was obtained from the area of late enhancement and in 19 active myocarditis was found.

In the study of Yelgec et al. [16], among 20 patients with acute myocarditis (mean LVEF=57%), delayed enhancement was present in 15 (75%) predominantly in the lateral LV wall (61%). In 15 of these 16 patients, there was a match between regional functional abnormalities and delayed enhancement.

Stensaeth et al. [17] found delayed enhancement in 27 of 42 patients with myocarditis (64%). In this series pericardial effusion occurred in 7 patients (17%). Late gadolinium enhancement occurred predominantly, in 86%, in the infero-lateral segments in mid or subepicardial segments.

Goitein et al. [18] published a series of 23 patients with acute myocarditis (mean LVEF=57%). Delayed enhancement was found in 21 of 23 patients (91%) in the infero-lateral region of the LV mostly in mid-ventricular segments. In this study echo detected wall motion abnormalities in 8 of 23 patients (35%). The distribution of wall motion abnormalities on echo matched the distribution of myocardial delayed enhancement on MRI in 6 of the 8 patients in whom wall motion abnormalities were seen on echo. In this study wall motion abnormalities seen on echo most often involved the inferior and infero-lateral segments at the mid LV.

Left ventricular dysfunction in myocarditis had been described previously in old echocardiographic studies. Of 41 patients with biopsy proven myocarditis 24 [58%] had ejection fraction less than 50%; 25 [64%] had regional LV dysfunction more often localized at the interventricular septum and at the apex; clinical presentation in most of the patients were congestive heart failure or arrhythmias [19]. This patient population was different from our patients. Today we know, that significant LV dysfunction and septo-apical wall motion abnormalities may be consistent with HHV-6 infection [7].

In another study published in 1984 [20], regional LV dysfunction was found in all 68 patients with myocarditis with predominant involvement of infero-apical segments. In this study M-mode echo technique was used.

In our study, wall motion abnormalities involved predominantly the infero-postero-lateral segments, and these observations are in concordance with results of MRI studies, dercribed above. These findings are also strongly supported by the postmortem study of Shirani et al. [21] in 1993, who found predominant location of gross myocardial lesions in acute mononuclear-cell myocarditis in the subepicardial region of the left ventricular free wall.

Long-term follow-up (mean 37 months) demonstrated that the outcome of patients with acute inflammatory peri-myocardial diseases is benign, however recurrent hospitalization due to recurrent inflammation is rather frequent.

### Limitations

1. MRI was not a routine practice in our hospital in perimyocarditis during the study period.

2. This is a retrospective study, based on hospital records, however echocardiographic images, electrocardiograms and medical histories were all reviewed thoroughly.

3. An 18-segments model of the left ventricle was used instead of the traditional 16 –segments model. This was based on our previous experience published earlier.

## Conclusions

In spite of these limitations, we believe that these data indicate that patients with acute inflammatory pericardial diseases often have regional infero-postero-lateral myocardial dysfunction that can be detected by conventional echo. Speckle tracking imaging and in particular late enhancement by MRI may provide additional important information. Recurrent hospitalization due to inflammation is rather frequent, however long term outcome appears to be benign.

## Abbreviations

LV: Left ventricle; MRI: Magnetic resonance imaging; Tn: Troponin; CK: Creatinin phosphokinase; CRP: C-reactive protein; ESR: Erythrocyte sedimentation ratio; EF: Ejection fraction; CT: Computerized tomography.

## Competing interests

The authors declare that they have no competing interests.

## Authors’ contributions

ML: study design, collection and analysis of data, conclusions, review of literature and writing the manuscript. VT: analysis and presentation of results, statistical assessment. EP: intellectual input, analysis of data. LC: intellectual input, analysis of data. ZV: study design, analysis of data, discussion of results and conclusions, writing the manuscript and final approval. All authors read and approved the final manuscript.

## Supplementary Material

Additional file 1 **Video.** Mid postero-lateral and mid inferior hypokinesis in the patients with mild myocarditis. Click here for file
